# Sequence dependent effect of paclitaxel on gemcitabine metabolism in relation to cell cycle and cytotoxicity in non-small-cell lung cancer cell lines

**DOI:** 10.1054/bjoc.2000.1399

**Published:** 2000-10

**Authors:** J R Kroep, G Giaccone, C Tolis, D A Voorn, W J P Loves, C J van Groeningen, H M Pinedo, G J Peters

**Affiliations:** Department of Medical Oncology, University Hospital Vrije Universiteit, P.O Box 7057, Amsterdam, MB, 1007, The Netherlands

**Keywords:** gemcitabine, paclitaxel, NSCLC cell lines, cell cycle, cytotoxicity, dFdCTP

## Abstract

Gemcitabine and paclitaxel are active agents in the treatment of non-small-cell lung cancer (NSCLC). To optimize treatment drug combinations, simultaneously and 4 and 24 h intervals, were studied using DNA flow cytometry and multiple drug effect analysis in the NSCLC cell lines H460, H322 and Lewis Lung. All combinations resulted in comparable cytotoxicity, varying from additivity to antagonism (combination index: 1.0–2.6). Gemcitabine caused a S (48%) and G1 (64%) arrest at IC-50 and 10 × IC-50 concentrations, respectively. Paclitaxel induced G2/M arrest (70%) which was maximal within 24 h at 10 × IC-50. Simultaneous treatment increased S-phase arrest, while at the 24 h interval after 72 h the first drug seemed to dominate the effect. Apoptosis was more pronounced when paclitaxel preceded gemcitabine (20% for both intervals) as compared to the reverse sequence (8%, *P* = 0.173 for the 4 h and 12%, *P* = 0.051 for the 24 h time interval). In H460 cells, paclitaxel increased 2-fold the accumulation of dFdCTP, the active metabolite of gemcitabine, in contrast to H322 cells. Paclitaxel did not affect deoxycytidine kinase levels, but ribonucleotide levels increased possibly explaining the increase in dFdCTP. Paclitaxel did not affect gemcitabine incorporation into DNA, but seemed to increase incorporation into RNA. Gemcitabine almost completely inhibited DNA synthesis in both cell lines (70–89%), while paclitaxel had a minor effect and did not increase that of gemcitabine. In conclusion, various gemcitabine–paclitaxel combinations did not show sequence dependent cytotoxic effects; all combinations were not more than additive. However, since paclitaxel increased dFdCTP accumulation, gemcitabine incorporation into RNA and the apoptotic index, the administration of paclitaxel prior to gemcitabine might be favourable as compared to reversed sequences. © 2000 Cancer Research Campaign


British Journal of Cancer (2000) 83(8), 1069–1076
© 2000 Cancer Research Campaign
doi: 10.1054/ bjoc.2000.1399, available online at http://www.idealibrary.com on IDEAL®

### Sequence dependent effect of paclitaxel on gemcitabine metabolism in relation to cell cycle and cytotoxicity in non-small-cell lung cancer cell lines

**JR Kroep, G Giaccone, C Tolis, DA Voorn, WJP Loves, CJ van Groeningen, HM Pinedo and GJ Peters**

Department of Medical Oncology, University Hospital Vrije Universiteit, P.O Box 7057, 1007 MB Amsterdam, The Netherlands

**Summary** Gemcitabine and paclitaxel are active agents in the treatment of non-small-cell lung cancer (NSCLC). To optimize treatment drug combinations, simultaneously and 4 and 24 h intervals, were studied using DNA flow cytometry and multiple drug effect analysis in the NSCLC cell lines H460, H322 and Lewis Lung. All combinations resulted in comparable cytotoxicity, varying from additivity to antagonism (combination index: 1.0–2.6). Gemcitabine caused a S (48%) and G1 (64%) arrest at IC-50 and 10 × IC-50 concentrations, respectively. Paclitaxel induced G2/M arrest (70%) which was maximal within 24 h at 10 × IC-50. Simultaneous treatment increased S-phase arrest, while at the 24 h interval after 72 h the first drug seemed to dominate the effect. Apoptosis was more pronounced when paclitaxel preceded gemcitabine (20% for both intervals) as compared to the reverse sequence (8%, $P = 0.173$ for the 4 h and 12%, $P = 0.051$ for the 24 h time interval). In H460 cells, paclitaxel increased 2-fold the accumulation of dFdCTP, the active metabolite of gemcitabine, in contrast to H322 cells. Paclitaxel did not affect deoxycytidine kinase levels, but ribonucleotide levels increased possibly explaining the increase in dFdCTP. Paclitaxel did not affect gemcitabine incorporation into DNA, but seemed to increase incorporation into RNA. Gemcitabine almost completely inhibited DNA synthesis in both cell lines (70–89%), while paclitaxel had a minor effect and did not increase that of gemcitabine. In conclusion, various gemcitabine–paclitaxel combinations did not show sequence dependent cytotoxic effects; all combinations were not more than additive. However, since paclitaxel increased dFdCTP accumulation, gemcitabine incorporation into RNA and the apoptotic index, the administration of paclitaxel prior to gemcitabine might be favourable as compared to reversed sequences. © 2000 Cancer Research Campaign

**Keywords:** gemcitabine; paclitaxel; NSCLC cell lines; cell cycle; cytotoxicity; dFdCTP

Gemcitabine and paclitaxel are among the most active agents in the treatment of non-small-cell lung cancer (Giaccone, 1995; Rajkumar and Adjei, 1998). The rationale for the gemcitabine–paclitaxel combination is supported by their different mechanisms of action and the partially non-overlapping toxicities (Rowinsky et al, 1995; Van Moorsel et al, 1997). Understanding of possible drug–drug interactions and cell cycle disturbances induced by gemcitabine and paclitaxel may help designing appropriate treatment schedules.

Gemcitabine, a deoxycytidine analogue, is phosphorylated to its monophosphate by deoxycytidine kinase (dCK) and subsequently by nucleotide kinases to its active metabolite, gemcitabine triphosphate (dFdCTP) (Heinemann et al, 1988; Ruiz van Haperen et al, 1994). dFdCTP accumulation was clearly correlated to its cytotoxicity (Van Moorsel et al, 2000). Several self-potentiating mechanisms have been described (Heineman et al, 1995; Peters and Ackland, 1996), which may enhance the incorporation of dFdCTP into the DNA and possibly also into RNA. Gemcitabine induces a G0/G1 and S phase arrest (Hertel et al, 1990; Tolis et al, 1999) and triggers apoptosis in human leukaemia (Bouffard and Momparler, 1995; Huang and Plunkett, 1995) and solid tumour cells (Tolis et al, 1999).

*Received 2 March 2000*
*Revised 22 June 2000*
*Accepted 28 June 2000*

*Correspondence to:* GJ Peters

Paclitaxel acts as a mitotic spindle poison by blocking eukaryotic cells in the G2/M mitotic phase of the cell cycle (Schiff et al, 1979; Rowinsky et al, 1988). Paclitaxel promotes microtubule assembly and stabilization by preventing depolymerization, leading to inhibition of cell proliferation and induction of cell death (Schiff et al, 1979). At low concentrations paclitaxel inhibited mitosis by altering microtubule dynamics (Jordan et al, 1993), resulting in cells with abnormal size and DNA content causing cell death (Torres and Horwitz, 1998). At higher concentrations, paclitaxel caused formation of stable bundles of microtubules and increased microtubule polymer mass (Jordan et al, 1993), resulting in G2/M phase arrest and apoptosis (Torres and Horwitz, 1998).

Because of the different effects of each drug on cellular metabolism and cell cycle distribution, sequential drug administration may result in potentiation of both single agents. Gemcitabine showed synergism/additivity when combined with cisplatin, etoposide, mitomycin C and topotecan (Van Moorsel et al, 1999; Tolis et al, 1999). However, previously, the combination with paclitaxel was reported to be less than additive (Theodossiou et al, 1998). For paclitaxel, a marked resistance has been shown when cells are not cycling (Hahn et al, 1993; Theodossiou et al, 1998). Combinations of paclitaxel with alkylators or cisplatin were sequence dependent, showing superior cytotoxicity when paclitaxel was administered first (Rowinsky et al, 1993; Liebmann et al, 1994). In patients, we recently demonstrated that paclitaxel increased the accumulation of dFdCTP (Kroep et al, 1999), indicating that paclitaxel administration prior to gemcitabine might be favourable, as compared to its reverse sequence.

To elucidate potential mechanisms for paclitaxel and gemcitabine interactions we investigated metabolic drug–drug interactions and used different sequences to determine the role of cell cycle effects on cytotoxicity of the combinations.

### MATERIAL AND METHODS

### Chemicals

Paclitaxel (Taxol®; Bristol-MyersSquibb, Princeton, NY) was dissolved in 100% ethanol. Gemcitabine (Gemzar®; 2′,2′-difluoro-2′-deoxycytidine; dFdC) and (5-$^3$H)-gemcitabine (16.7 Ci/mmol) were a kind gift of Eli Lilly Inc., Indianapolis, USA and were dissolved in phosphate buffered saline (PBS) and 50% (v/v) ethanol, respectively. (8-$^3$H)-2-chlorodeoxyadenosine ($^3$H-CdA, 24.2 Ci/mmol) was purchased from Moravek Brea, CA, USA, (2-$^{14}$C)-Thymidine ($^{14}$C-TdR, 59.7 Ci/mmol) from Dupont de Nemours NEND, Boston, MA, USA and (5-$^3$H)-uridine ($^3$H-UR, 27.0 Ci/mmol) from Amersham International, Buckinghamshire, England. All other chemicals were of analytical grade.

### Cell culture

The NSCLC cell lines Lewis Lung carcinoma (LL, murine) [Mayo, 1972], H460 (NCl-H460, human, wild-type-p53) and H322 (NCl-H322, human, mutant-p53) (Winter et al, 1992) were grown in monolayers and maintained in exponential growth in RPMI 1640 supplemented with 10% heat-inactivated fetal calf serum (FCS) (GIBCO, Paisley, UK) and 250 ng/ml gentamycin at 37°C at 5% carbon dioxide. Cell doubling times were 26 h, 18 h and 40 h under these conditions, respectively.

### Growth inhibition assay

Antiproliferative effects were analysed by the sulforhodamine B assay as described previously (Keepers et al, 1991). Briefly, cells were seeded in triplicate in 96-well flat bottom plates (Costar, Cambridge, MA, USA): LL, 5000 cells/well; H460, 5000 cells/well and H322, 20 000 cells/well. After 24 h drugs were added with a gemcitabine:paclitaxel ratio of 1:3, based on the obtained IC-50 values. Cell lines were exposed for 4, 24 and 72 h, with a total culture time of 96 h. In H460 cells, sequential drug exposure, gemcitabine 4 and 24 h before paclitaxel and the reverse sequences, were also tested.

In order to quantify the degree of drug interaction, multiple drug effect analysis was performed based on that described by Chou and Talalay (Chou and Talalay, 1984) using computer software of Chou (CalcuSyn v 1.1.1, Biosoft 1996, Cambridge, UK). Results were expressed as mutually non-exclusive combination index (CI) values for every fraction affected (FA), while for the final evaluation we used the averaged CI at 0.5, 0.75, 0.90, and 0.95 FA, representing relevant growth inhibition values. In the current software package (Calcusyn), the classification for the effect is as follows: CI < 0.3 = highly synergistic; 0.3–0.7 = synergistic; 0.7–0.9 = moderate to slight synergistic; 0.9–1.1 = nearly additive; 1.1–1.45 = slight to moderate antagonistic, 1.45–3.3 = antagonistic; >3.3 = strong antagonistic.

### Flow cytometry

To determine the cell cycle events following different gemcitabine–paclitaxel combinations, flow cytometric measurement of cellular DNA content using propidium iodide (PI) (Sigma Aldrich, Deisenhofen, Germany) was performed as described previously (Tolis et al, 1999). Briefly, H460 and H322 cells were exposed to gemcitabine and paclitaxel alone and simultaneously at IC-50 and 10 × IC-50 concentrations. At 4, 24, 48 and 72 h after start of drug exposure, cells (including the floating cells) were harvested, resuspended in PI solution and analysed on a FACScan flow cytometer (Beckton Dickinson, Mountain View, CA, USA), using the cell fit program (CellFit™, Becton Dickinson, San Jose, CA). Cells which were less intensively stained than G1 cells (sub-G1 cells), were considered apoptotic cells. The flow rate was at about 200–500 nuclei/s and at least $20 \times 10^4$ cells of each sample were analysed. Measurements were performed under the same instrumental settings. Based on these experiments, the 4 and 24 h time-intervals were chosen for sequential drug exposure. These latter cultures were incubated for 28, 48 and 72 h.

### The effect of paclitaxel on dCK activity

To determine a possible influence of paclitaxel on dCK activity, H460 and H322 cells were exposed to paclitaxel at IC-50 for 24 h, after which cells were harvested as described previously (Ruiz van Haperen et al, 1996), and stored at –80°C until analysis. dCK was assayed essentially as described previously (Ruiz van Haperen et al, 1996) but utilizing (8- $^3$H)-2-chlorodeoxyadenosine ($^3$H-CdA; 50 µM; 0.16 µCi/nmol) as the substrate (Spasokoukotskaja et al, 1995). Enzyme activities were expressed as nmol product formed per h per $10^6$ cells (nmol/h/$10^6$ cells).

### dFdCTP accumulation

To determine the influence of paclitaxel on the dFdCTP accumulation, cells were seeded in 2 ml 6-well plates (H460, $2.5 \times 10^5$ cells/well; H322, $5 \times 10^5$ cells/well). After 24 h, cells were exposed to gemcitabine at 0.1 and 1.0 µM with or without paclitaxel at 0.033 and 0.33 µM, respectively, for 24 h at 37°C. Cells were harvested, nucleotides were extracted and dFdCTP and nucleotides were analysed by HPLC as described previously (Ruiz van Haperen et al, 1994).

### Determination of the influence of paclitaxel on gemcitabine incorporation

Incorporation of $^3$H-gemcitabine into DNA and RNA was performed essentially as described previously (Van Moorsel et al, 1999). Briefly, cells were plated ($1 \times 10^4$ H460 and $2 \times 10^4$ H322 cells) into 96-well filter bottom plates (Multiscreen® Filtration system, 0.22 µm Hydrophilic Low Protein Binding Durapore® membrane, Millipore, Ettenleur, The Netherlands), and after 24 h exposed to (5-$^3$H)-gemcitabine 0.05 µM final concentration (21 Ci/mmol) with or without 0.17 or 0.017 µM paclitaxel for 4 and 24 h at 37°C. Incorporation into DNA was determined by adding 20 µl RNAase A/T1 (500 U/ml; DNAase-free) and 80 µl PBS to one part of the wells followed by an incubation for 30 min at 37°C. RNA incorporation was determined by subtracting the DNA incorporation from the total incorporation. Inhibition of DNA and RNA synthesis was measured in separate cultures

 © 2000 Cancer Research Campaign

**Table 1** Sensitivity of NSCLC cell lines for gemcitabine and paclitaxel

| Cell line | IC-50 Gemcitabine (nM) | | | IC-50 Paclitaxel (nM) | | |
|---|---|---|---|---|---|---|
| | 4 h | 24 h | 72 h | 4 h | 24 h | 72 h |
| H460 | 160 ± 7 | 28 ± 1 | 4.3 ± 3.7 | 680 ± 169 | 141 ± 60 | 88 ± 32 |
| H322 | 708 ± 335 | 420 ± 201 | 25 ± 13 | 1900 ± 893 | 14 ± 4 | 11 ± 6 |
| LL | 800 ± 100 | 27 ± 7 | 13 ± 4 | 410 ± 90 | 26 ± 12 | 5.6 ± 3.0 |

The IC-50 is defined as the concentration causing 50% growth inhibition in treated cells after 4, 24 and 72 h exposure to each drug alone, followed by 68, 48 and 0 h incubation in drug free medium, respectively, as compared to control cells. Values are means ± SEM of at least three separate experiments.

exposed to the drugs by measuring the incorporation of $^{14}$C-TdR (59.7 mCi/mmol; 5.6 μM) and $^{3}$H-UR (27 Ci/mmol; 165 nM) for the last 2 h of the assay.

### RESULTS

### Growth inhibition of gemcitabine and paclitaxel

Table 1 summarizes the sensitivities of the three NSCLC cell lines to gemcitabine and paclitaxel. H460 cells were most sensitive to gemcitabine, but were the least sensitive to paclitaxel after 24 and 72 h exposure. For both drugs IC-50 values after 72 h exposure were lower than after shorter incubation times. Based on these data, combination ratios were designed for simultaneous and sequential combinations. Representative growth inhibition curves in LL cells are shown in Fig. 1a.

Fig. 1b depicts the multiple drug-effect analysis for LL cells. The CI/FA plot after 4 h drug exposure showed antagonism at the lower FA, while at the more relevant FA (≥50%) an additive effect was observed. The average CI values for gemcitabine and paclitaxel combined in LL, H460 and H322 cells are summarized in Table 2. Simultaneous drug administration resulted in an additive to antagonistic effect, represented by a CI ranging from 1.0 to 2.6. Based on the cell cycle effects of both gemcitabine and paclitaxel (see next section) we reasoned that sequential drug treatment would exploit cell cycle arrest caused by one drug in order to increase sensitivity to the other drug. These schedule dependent drug interactions were investigated in the H460 cell line (Table 2). For all schedules the effect was not more than additive.

### Effects on the cell cycle

DNA flow cytometry studies were performed to determine the effect of different gemcitabine–paclitaxel combinations on the cell cycle distribution and to determine whether these could be used to optimize scheduling. Gemcitabine at IC-50 concentrations caused accumulation of cells in the S-phase, while at 10 × IC-50 concentrations (Fig. 2) cells predominantly accumulated in G0/G1 phase. Paclitaxel at IC-50 concentrations increased accumulation in the G2/M phase, which was maximal within 24 h and more pronounced at 10 × IC-50 values (Fig. 2). At 24 h, simultaneous exposure to gemcitabine and paclitaxel resulted in an accumulation of the cells in the S phase of the cell cycle at the 10 × IC-50 concentrations (45%).

For the study of sequential applications, gemcitabine was added 4 and 24 h prior to or after paclitaxel and cultures were incubated up to 28, 48 and 72 h (Table 3). Exposure to gemcitabine 4 or 24 h

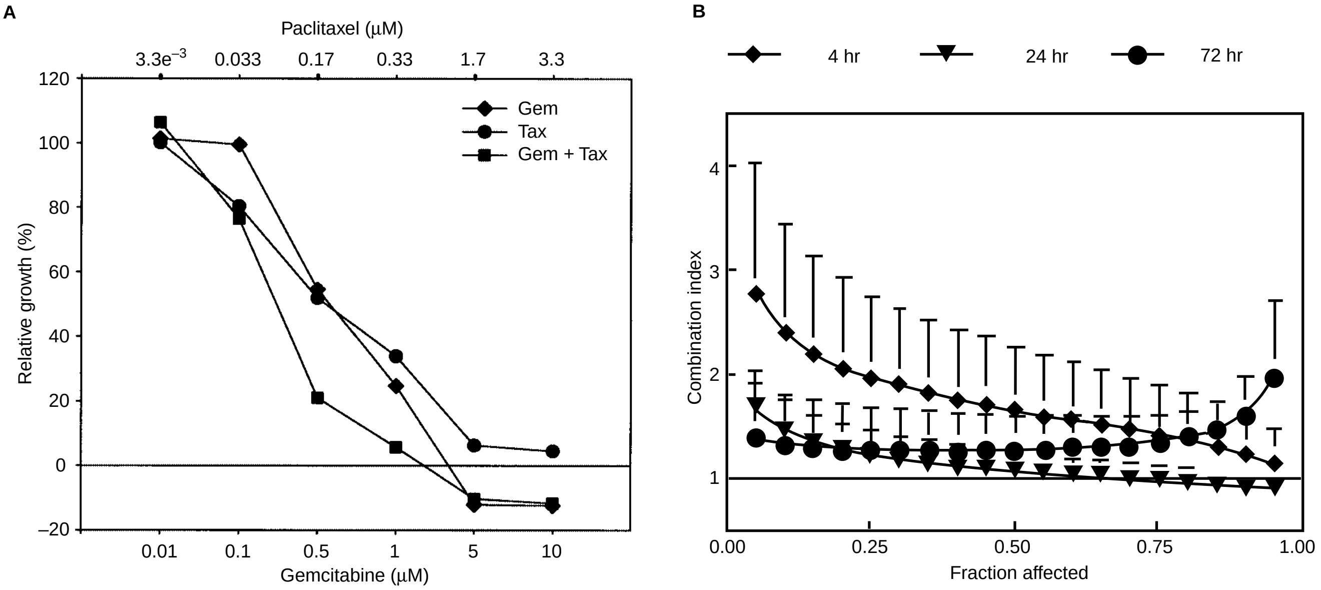

**Figure 1** (**A**) Representative growth inhibition curves of gemcitabine (◆) and paclitaxel (●) alone and in combination (■) in LL cells. Cells were exposed to the drugs for 4 h followed by 68 h drug free medium, and growth inhibition was determined after 72 h by the sulforhodamine B assay. (**B**) Multiple drug effect analysis of the interaction between gemcitabine and paclitaxel in LL cells exposed for 4 h (●), 24 h (▼) and 72 h (■) to the combination. Results are th mean ± SEM of at least 3 experiments

*© 2000 Cancer Research Campaign*

**Table 2** Evaluation of synergism/antagonism with the multiple drugs effect analysis

| Drug exposure | LL | H460 | H322 |
|---|---|---|---|
| Simultaneous | | | |
| 4 h | 1.4 ± 0.4 | 2.6 ± 1.0 | 1.4 ± 0.4 |
| 24 h | 1.0 ± 0.1 | 1.8 ± 0.3 | 2.0 ± 0.8 |
| 72 h | 1.5 ± 0.3 | 1.5 ± 0.3 | 1.1 ± 0.8 |
| Sequentially | | | |
| Gem > 4 hr > Tax | | 1.5 ± 0.2 | |
| Gem > 24 hr > Tax | | 1.3 ± 0.2 | |
| Tax > 4 hr < Gem | | 1.6 ± 0.3 | |
| Tax > 24 hr > Gem | | 1.0 ± 0.1 | |

Mean combination index (CI) values after exposure to gemcitabine and paclitaxel simultaneously for 4, 24 and 72 h in LL, H460 and H322 cells and sequentially, 4 and 24 h intervals, in H460 cells for 72 h. The average CI values are the mean of 0.5, 0.75, 0.90 and 0.95 FA. Values are means ± SEM of at least 3 separate experiments.

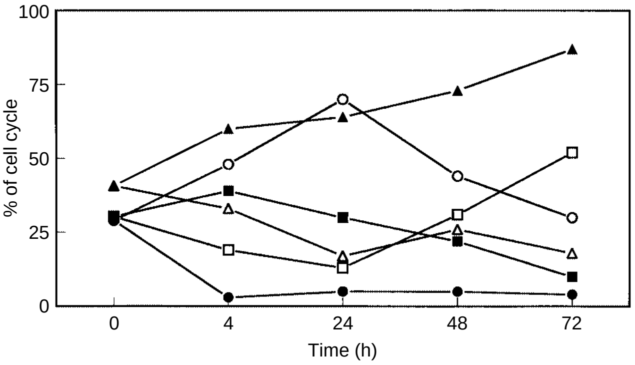

**Figure 2** Cell cycle distribution – time curve for gemcitabine (closed symbols) and paclitaxel (open symbols) alone at 10 × IC-50 concentration in H460 NSCLC cells. The percentage of cells in the G0/G1 (triangles), S (squares) and G2/M phases (circles) of the cell cycle are mean values of 3 or 4 experiments with SEM ≤ 28 of the mean prior to paclitaxel resulted in an increased G2/M arrest after 28 h as compared to exposure to gemcitabine alone, comparable to that of paclitaxel alone. For the reversed sequences, the second drug, gemcitabine, also dominated the cell cycle effect at 28 h. However, after 72 h, the first drug seemed to dominate for both sequences, with a significantly different cell cycle distribution for the 24 h time interval (Table 3). From 24 until 72 h the fraction of arrested cells decreased rapidly, while the fraction of dead cells (either apoptotic or necrotic cells) increased proportionally.

### Cell death

Flow cytometric analysis of cell death using PI staining permits simple measurement of apoptosis (Nicoletti et al, 1991). Previously, we compared three methods for assessment of the apoptotic index: analysis of morphological changes using May-Grünwald-Giemsa (MGG) staining, the TUNEL assay and FACS analysis (Tolis et al, 1999). Since relative effects were comparable, we used FACS analysis to determine the sub-G1 peak for assessment of the influence of drug scheduling on apoptotis. Basal levels of apoptosis ranged from 2.5 ± 0.4% after 4 h to 4.4 ± 1.5% after 72 h, which increased after treatment (Fig. 3). Apoptotic cell death at 72 h was significantly higher ($P = 0.05$) when paclitaxel was added 24 h prior to gemcitabine (20% ± 4) as compared to the reverse sequence (12% ± 5). Combined drug treatment also induced necrosis time- and concentration-dependently; after 72 h ranging from 35 to 40% at IC-50 concentrations and from 67 to 72% at 10 × IC50 concentrations. No schedule dependent differences were observed.

### Effect on dFdCTP accumulation and normal nucleotide pools

In mononuclear cells of patients, we previously observed a paclitaxel induced increase of dFdCTP, the main metabolite of gemcitabine (Kroep et al, 1999). Therefore, we measured the accumulation of dFdCTP in NSCLC cells. In H460 cells, 0.03 μM paclitaxel significantly ($P = 0.035$) increased dFdCTP accumulation from 28 ± 8 to 62 ± 2 pmol/$10^6$ cells after exposure to 0.1 μM

**Table 3** Effect of gemcitabine–paclitaxel sequences on cell cycle distribution in H460 cells

| Drugs | Cell cycle phase | 28 h | 48 h | 72 h |
|---|---|---|---|---|
| Gem + Tax | G0/G1 | 38 ± 9[×] | 46 ± 0.7 | 62 ± 0.5 |
| | S | 26 ± 5[×] | 28 ± 0.8 | 24 ± 0.7 |
| | G2/M | 37 ± 4[×] | 26 ± 0.1 | 15 ± 1 |
| Gem > 4 h > Tax | G0/G1 | 33 ± 5 | 49 ± 3[❖] | 57 ± 6[#] |
| | S | 28 ± 2 | 30 ± 3 | 27 ± 4 |
| | G2/M | 40 ± 4 | 21 ± 1[❖] | 16 ± 3[#❖] |
| Gem > 24 h > Tax | G0/G1 | 25 ± 5[#] | 40 ± 4[❖] | 61 ± 5[#❖] |
| | S | 40 ± 2 | 34 ± 7 | 26 ± 2[❖] |
| | G2/M | 35 ± 4 | 29 ± 3 | 13 ± 3[#❖†] |
| Tax > 4 h > Gem | G0/G1 | 38 ± 4 | 48 ± 3 | 45 ± 5 |
| | S | 37 ± 8 | 30 ± 3 | 33 ± 3 |
| | G2/M | 28 ± 4 | 22 ± 1 | 23 ± 3 |
| Tax > 24 h > Gem | G0/G1 | 48 ± 4* | 50 ± 4 | 43 ± 6* |
| | S | 30 ± 2* | 28 ± 2 | 34 ± 3* |
| | G2/M | 23 ± 4* | 22 ± 3* | 25 ± 3 |

Cell cycle distributions in H460 cells exposed to gemcitabine (Gem) simultaneously and 4 and 24 h prior to paclitaxel (Tax) and the reverse sequences at IC-50 concentrations, as determined by FACS analysis using PI staining. Cultures were incubated up to [×]24 (simultaneous), 28, 48 and 72 h. The cell cycle distribution at 0 h was as follows: G1/G0: 41 ± 3, S: 30 ± 5, G2/M: 29 ± 4. Values are means ± SEM of 3 experiments. *Significantly different from the reverse sequence ($P < 0.05$). [#]Significantly different from untreated cells ($P < 0.05$). [❖]Significantly different from 28 hr ($P < 0.03$). [†]Significantly different from 48 hr ($P = 0.01$).

*© 2000 Cancer Research Campaign*

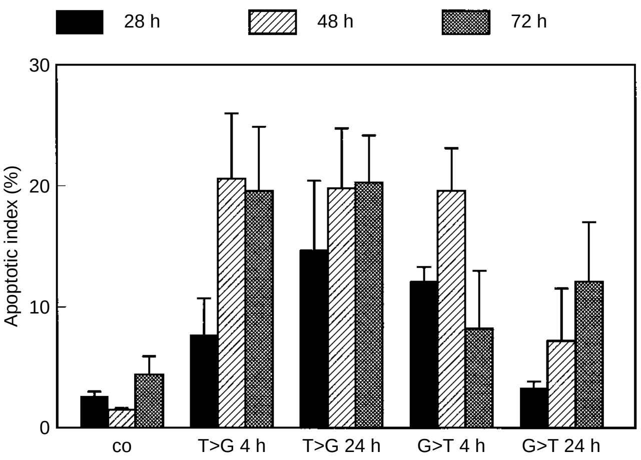

**Figure 3** The percentage of apoptotic cells, defined as apoptotic index, for the four schedules, gemcitabine (G) 4 and 24 h prior to paclitaxel (T) and both reversed schedules, after 28 (solid bars), 48 (hatched bars) and 72 h (double hatched bars) of incubation. Apoptosis (sub-G1 peak) was determined by FACS analysis using PI staining. The apoptotic index of gemcitabine alone was 3.3 ± 0.8, 1.9 ± 0.6 and 6.0 ± 1.3 after 28, 48 and 72 h, respectively; the apoptotic index of paclitaxel 6.8 ± 3.1, 10.7 ± 4.2 and 15.8 ± 2.9, respectively. Values are means ± SEM of 3 experiments gemcitabine (Table 4). At 1.0 µM gemcitabine dFdCTP accumulation was 370 ± 87 pmol/$10^6$ cells, but 0.3 µM paclitaxel only moderately increased dFdCTP accumulation to 429 ± 78 pmol/$10^6$ cells. In H322 cells, dFdCTP accumulation at 0.1 and 1.0 µM gemcitabine was 67 ± 5 and 462 ± 55 pmol/106 cells, but paclitaxel did not increase dFdCTP accumulation.

Increased dFdCTP accumulation might be related to altered metabolism of cofactors involved in the synthesis of dFdCTP, such as ATP and UTP, cofactors in gemcitabine phosphorylation, while CTP and UTP may also regulate dCK (Ruiz van Haperen et al, 1996). In the NSCLC cells all ribonucleotide levels clearly increased after gemcitabine alone and the gemcitabine-paclitaxel combination (Table 4), in accordance with the results previously obtained in vitro and in patients (Kroep et al, 1999; Van Moorsel et al, 2000). In H322 cells the increase for all ribonucleotides was less pronounced, maximal 1.8 fold.

In order to determine a possible role of dCK in the increased dFdCTP accumulation, dCK activity was measured after 24 h incubation with or without paclitaxel (IC-50) in the H460 and H322 cell lines. No differences in dCK activity were found between exposed and non-exposed cells. Furthermore, in H460 and H322 control cells, the dCK activities were similar, 0.8 ± 0.1 and 0.7 ± 0.1 nmol/h/$10^6$ (mean ± SEM), respectively.

### Gemcitabine incorporation into DNA and RNA

The action of gemcitabine is dependent on its incorporation into DNA and possibly into RNA, resulting in inhibition of nucleic acid synthesis. We determined whether paclitaxel affected the gemcitabine incorporation into DNA and RNA and its influence on inhibition of DNA and RNA synthesis in the H460 and H322 cell lines. Gemcitabine clearly inhibited the DNA synthesis after 4 and 24 h of drug exposure in H460 cells, while in H322 cells inhibition was more pronounced (Table 5). Paclitaxel alone did not inhibit the DNA synthesis after 4 h exposure, but after 24 h exposure a moderate inhibition was observed. However, in both cell lines the drug combination did not increase the inhibition of nucleic acid synthesis after 4 and 24 h exposure, as compared to gemcitabine alone (Table 5). The latter might explain the additive effect of the gemcitabine and paclitaxel combination.

Gemcitabine incorporation into DNA and RNA was drug exposure- and time-dependent (Table 6); gemcitabine incorporation into DNA increased almost 2-fold after 24 h exposure, as compared to 4 h. In both H460 and H322 cells, paclitaxel did not affect the gemcitabine incorporation into DNA. Paclitaxel seemed to stimulate the gemcitabine incorporation into RNA (Table 5), especially in H460 cells. Overall, gemcitabine incorporation into RNA was lower than that into DNA. Gemcitabine incorporation into DNA was 2 fold higher in the most sensitive cell line H460 as compared to H322.

### DISCUSSION

Scheduling of treatment plays an important role in optimizing the efficacy of a drug combination. In NSCLC cells, paclitaxel administration prior to gemcitabine induced a higher apoptotic index and might therefore be more favourable than the reverse sequence. Although the precise cell killing mechanisms of gemcitabine and paclitaxel are still unresolved, apoptosis plays a role in cell death for both agents (Huang and Plunkett, 1995; Jordan et al, 1996). Apoptosis was more pronounced in the schedules in which paclitaxel preceded gemcitabine, which might be explained by direct drug effects as well as downstream signalling effects. Paclitaxel has a high apoptotic index (Fig. 3) (Jordan et al, 1996) as

**Table 4** Effect of gemcitabine and paclitaxel combinations on ribonucleotide levels in H460 NSCLC cells

| Drug | Ribonucleotides; relative to pretreatment levels [mean ± SEM (*P*-value)] | | | |
|---|---|---|---|---|
| | CTP | UTP | ATP | GTP |
| Gemcitabine 0.1 µM | 2.2 ± 0.4[b] | 1.8 ± 0.3[b] | 1.6 ± 0.3[b] | 1.6 ± 0.3[c] |
| Gemcitabine 0.1 µM + paclitaxel 0.03 µM | 2.2 ± 0.3[a] | 1.9 ± 0.3[b] | 1.8 ± 0.2[a] | 1.6 ± 0.1[a] |
| Gemcitabine 1.0 µM | 2.0 ± 0.4 | 2.0 ± 0.5[c] | 1.7 ± 0.3[c] | 1.5 ± 0.2[c] |
| Gemcitabine 1.0 µM + paclitaxel 0.3 µM | 2.0 ± 0.5[c] | 2.1 ± 0.5[c] | 1.9 ± 0.5[c] | 2.0 ± 0.4[c] |

Ribonucleotide levels were measured in H460 cells after 24 h exposure to gemcitabine plus or minus paclitaxel, respectively. Pretreatment nucleotide levels were set at 1% for each experiment. Ribonucleotide levels (mean ± SEM; pmol/$10^6$ cells) at t = 0 were as follows: CTP = 215 ± 74, UTP = 1058 ± 437, ATP = 1206 ± 547, GTP = 443 ± 185. *Significantly different from gemcitabine alone (*P* = 0.020). a = $P \leq 0.001$; b = $P \leq 0.01$; c = $P \leq 0.05$.

© 2000 Cancer Research Campaign

**Table 5** Inhibition of DNA and RNA synthesis by gemcitabine, paclitaxel and the combination in H460 and H322 cells

| Cell line | Incubation (h) | DNA synthesis (%) | | | RNA synthesis (%) | | |
|---|---|---|---|---|---|---|---|
| | | Gem | Tax | Gem + Tax | Gem | Tax | Gem + Tax |
| H460 | 4 | 30 ± 4 | 115 ± 12 | 28 ± 9 | 62 ± 10 | 124 ± 4 | 67 ± 6 |
| | 24 | 15 ± 3 | 61 ± 25 | 17 ± 4 | 70 ± 1 | 83 ± 14 | 73 ± 4 |
| H322 | 4 | 20 ± 11 | 147 ± 5 | 21 ± 12 | 79 ± 18 | 110 ± 2 | 82 ± 17 |
| | 24 | 11 ± 1 | 94 ± 3 | 15 ± 2 | 90 ± 28 | 134 ± 35 | 96 ± 29 |

The NSCLC cell lines H460 and H322 were exposed to 0.05 μM gemcitabine (Gem) alone, or in combination with 0.15 μM paclitaxel (Tax), for 4 and 24 h. The effect of the drugs on both DNA and RNA synthesis is given as percentage of TdR and UR incorporation without drugs. For H460 cells TdR incorporation without drugs at the end of the 4 and 24 h incubation was 41.4 ± 3 and 32.3 ± 5.9 pmol/$10^6$ cells, respectively; UR incorporation: 1.1 ± 0.1 and 0.75 ± 0.4 nmol/$10^6$ cells, respectively. For H322 cells TdR incorporation without drugs at the end of the 4 and 24 h incubation was 14.3 ± 4.7 and 23.1 ± 4.0 pmol/$10^6$ cells, respectively; UR incorporation: 0.59 ± 0.4 and 0.52 ± 0.1 nmol/$10^6$ cells, respectively. Values are means ± SEM of three separate experiments.

**Table 6** Effect of paclitaxel on gemcitabine incorporation into DNA and RNA of NSCLC cell lines

| Cell line | Incubation (h) | Incorporation into DNA | | Incorporation into RNA | |
|---|---|---|---|---|---|
| | | Gem alone | Gem + Tax | Gem alone | Gem + Tax |
| H460 | 4 | 33.3 ± 0.4 | 34.9 ± 5.9 | 3.4 ± 3.7 | 3.2 ± 5.6 |
| | 24 | 51.3 ± 13.3 | 42.6 ± 7.3 | 0.0 ± 0.0 | 12.1 ± 9.3 |
| H322 | 4 | 14.8 ± 5.8 | 13.3 ± 4.3 | 0.0 ± 0.0 | 0.0 ± 0.0 |
| | 24 | 29.0 ± 11.2 | 33.4 ± 11.2 | 0.4 ± 0.5 | 2.4 ± 3.7 |

H460 and H322 cells were exposed to 0.05 μM gemcitabine alone, or in combination with 0.15 μM paclitaxel, for 4 and 24 h. Values (fmol gemcitabine/$10^6$ cells) are means ± SEM of three separate experiments.

compared to gemcitabine, explaining the more pronounced apoptosis when paclitaxel is administered first and thus for a longer time. The increase in apoptosis may be related to increased dFdCTP levels, since dFdCTP causes a (deoxy)ribonucleotide pool imbalance and thereby contributes to induction of apoptosis (Plunkett and Huang, 1995; Van Moorsel et al, 2000). Moreover, the observed sequence dependent cell cycle distribution at 72 h may play a role; the difference in G2/M to G1/S ratio at the 24 h interval or the downstream factors directing the cells to G1/S or G2/M arrest. There is not yet sufficient evidence to conclude whether the assessment of apoptotic index is of prognostic value, but with some drugs, such as topotecan or paclitaxel, apoptosis appeared to correlate with the initial clinical response (Seiter et al, 1995).

Although the sequence paclitaxel before gemcitabine seemed more favourable, growth inhibition of the combination was not more than additive, irrespective of treatment schedule. This is in partial agreement with data of Theodossiou et al (1998), who found a less than additive effect when both agents were incubated simultaneously or sequentially in human lung, breast and pancreatic cancer cell lines. This might be related to the different effects on cell cycle distribution. Cells delayed by gemcitabine in G1/S phase cannot proceed through the cell cycle and will be less sensitive for the cytotoxic effects of paclitaxel. On the other hand, treatment with paclitaxel will first induce a G2/M block, which might decrease the gemcitabine effect. In addition, the lack of interaction between both agents at the DNA level might explain the no more than additive effect.

Mechanistic studies concentrated on gemcitabine metabolism, with emphasis on dFdCTP accumulation and gemcitabine incorporation into DNA and RNA. In accordance with the results obtained in patients (Kroep et al, 1999), paclitaxel increased dFdCTP accumulation in H460 cells. To get a better understanding of the mechanism for this phenomenon the influence on dCK and ribonucleotide levels was also studied. dCK can be activated by various genotoxic agents (Sasvari-Szekely et al, 1998, 1999), but in NSCLC cells paclitaxel failed to show a direct effect on dCK activity. However, the increased ribonucleotide levels in H460 cells might have contributed to gemcitabine phosphorylation, while the lower increase in H322 cells might explain the lack of increased dFdCTP levels. Despite the dFdCTP increase in H460 cells, paclitaxel did not influence the incorporation of gemcitabine into DNA of these cells or that of H322 cells. Interestingly, paclitaxel seemed to increase gemcitabine incorporation into RNA, although it is not clear how this relates to an increased apoptotic index. However, for another fluoropyrimidine, 5-fluorouracil, its incorporation into RNA was previously shown to be related to the extent of apoptosis (Pritchard et al, 1997). The extent of gemcitabine incorporation into DNA was related to the gemcitabine sensitivity, since DNA incorporation was higher in the most sensitive cell line H460.

In phase II trials in patients with NSCLC the combination of gemcitabine and paclitaxel appeared to be well-tolerated, with response rates ranging from 29% to 58% (Dombernowsky et al, 1998; Giaccone et al, 2000). Paclitaxel and gemcitabine have been evaluated in a phase I study using a biweekly schedule (Rothenberg et al, 1998), in which high doses of gemcitabine (3000 mg/m$^2$) could be given. However, the once weekly schedule of gemcitabine (800–1500 mg/m$^2$) administered as a 30 min infusion is generally accepted as the most active one. In combination, gemcitabine is usually preceded by paclitaxel as a 3 h infusion once (175–200 mg/m$^2$) or weekly (100–150 mg/m$^2$). The weekly administration of both drugs might enhance a potentiating interaction. Gemcitabine has also been given before paclitaxel (Pedersen, 1997), but our data support the more generally used paclitaxel before gemcitabine sequence. This combination is currently being

 *© 2000 Cancer Research Campaign*

compared in an ongoing randomized EORTC trial with the cisplatin-gemcitabine and the cisplatin-paclitaxel combinations.

In conclusion, although in vitro various schedules showed similar cytotoxicity, paclitaxel administration prior to gemcitabine seems to be favourable, because of the observed increased dFdCTP accumulation, gemcitabine incorporation into RNA and apoptotic index. Results of clinical studies may potentially be affected by the sequence in which these drugs are administered.

### ACKNOWLEDGEMENTS

This study was financially supported by Eli Lilly & Co, International and The Netherlands, and by the European Union (Biomed Grant BMH4 CT96–0479) and a European Cancer Centre grant to C Tolis.

### REFERENCES

Bouffard DY and Momparler RL (1995) Comparison of the induction of apoptosis in human leukemic cell lines by 2′,2′-difluorodeoxycytidine (gemcitabine) and cytosine arabinoside. *Leukemia Res* **19**: 849–856

Chou TC and Talalay P (1984) Quantitative analysis of dose–effect relationship: the combined effects of multiple drugs on enzyme inhibitors. In: *Advances in Enzyme Regulation*, G. Weber (ed.) pp.27–55. New York: Pergamon Press

Dombernowsky P, Giaccone G, Sandler A and Schwartsmann G (1998) Gemcitabine and paclitaxel combinations in non-small cell lung cancer. *Sem Oncol* **25** (Suppl 9): 44–50

Giaccone G (1995) New drugs in non-small cell lung cancer. An overview. *Lung Cancer* **12** (Suppl. 1): 155–162

Giaccone G, Smit EF, Van Meerbeeck JP, Splinter T, Golding RP, Pinedo HM, Laan D, Van Tinteren H and Postmus PE (2000) A phase I/II study of gemcitabine and paclitaxel in advanced non-small-cell lung cancer patients. *Ann Oncol* **11**: 109–112

Hahn SM, Liebmann JE, Cook J, Fisher J, Goldspiel B, Venzon D, Mitchell JB and Kaufman D (1993) Taxol in combination with doxorubicin or etoposide. Possible antagonism in vitro. *Cancer* **72**: 2705–2711

Heinemann V, Hertel LW, Grindey GB and Plunkett W (1988) Comparison of the cellular pharmacokinetics and toxicity of 2′,2′-difluorodeoxycytidine and 1-β-D-arabino-furanosylcytosine. *Cancer Res* **48**: 4024–4031

Heinemann V, Schultz L, Issels RD and Plunkett W (1995) Gemcitabine: a modulator of intracellular nucleotide and deoxynucleotide metabolism. *Sem Oncol* **22** (Suppl 11): 11–18

Hertel LW, Boder GB, Kroin JS, Rinzel SM, Poore GA, Todd GC and Grindey GB (1990) Evaluation of the antitumor activity of 2′,2′-difluoro-2′-deoxycytidine. *Cancer Res* **50**: 4417–4422

Huang P and Plunkett W (1995) Fludarabine and gemcitabine induced apoptosis: incorporation of analogues into DNA is a critical event. *Cancer Chemother Pharmacol* **36**: 181–188.

Jordan MA, Toso RJ, Thrower D and Wilson L (1993) Mechanism of mitotic block and inhibition of cell proliferation by taxol at low concentrations. *Proc Natl Acad Sci* **90**: 9552–9556

Jordan MA, Wendell K, Gardiner S, Derry B, Copp H and Wilson L (1996) Mitotic block induced in HeLa cells by low concentrations of paclitaxel (Taxol) results in abnormal mitotic exit and apoptotic cell death. *Cancer Research* **56**: 816–825.

Keepers YPAM, Pizao PE, Peters GJ, Van Ark-Otte J and Winograd B (1991) Comparison of the sulforhodamine B protein and tetrazolium (MTT) assays for *in vitro* chemosensitivity testing. *Eur J Cancer* **27**: 897–900

Kroep JR, Giaccone G, Voorn DA, Smit EF, Beijnen JH, Rosing H, Van Moorsel CJA, Van Groeningen CJ, Postmus PE, Pinedo HM and Peters GJ (1999) Gemcitabine and paclitaxel: pharmacokinetic and pharmacodynamic interactions in patients with non-small-cell lung cancer. *J Clin Oncol* **17**: 2190–2197

Liebmann JE, Fisher J, Teague D and Cook JA (1994) Sequence dependence of paclitaxel (Taxol) combined with cisplatin or alkylators in human cancer cells. *Oncol Res* **6**: 25–31

Mayo JG (1972) Biologic characterisation of the subcutaneously implanted Lewis Lung tumour. *Cancer Chem Rep* **21**: 325–330

Nicoletti I, Migliorati G, Pagliacci MC, Grignani F and Riccardi C (1991) A rapid and simple method for measuring thymocyte apoptosis by propidium iodide staining and flow cytometry. *J Immunol Meth* **139**: 271–279

Pedersen AG Phase I studies of gemcitabine combined with carboplatin or paclitaxel. *Sem Oncol* **Vol 24**(suppl 7): 64–68

Peters GJ and Ackland SP (1996) New anti-metabolites in preclinical and clinical development. *Exp Opin Invest Drugs* **5**: 637–679

Plunkett W and Huang P (1995) Fluarabine- and gemcitabine-induced apoptosis: incorporation of analogs into DNA is critical event. *Cancer Chem Pharm* **36**: 181–188

Pritchard DM, Watson AJM, Potten CS, Jackman AL and Hickman JA (1997) Inhibition by uridine but not thymidine of p53-dependent intestinal apoptosis initiated by 5-fluorouracil: evidence for the involvement of RNA perturbation. *Proc Natl Acad Sci* **94**: 1795–1799

Rajkumar SV and Adjei AA (1998) A review of the pharmacology and clinical activity of new chemotherapeutic agents in lung cancer. *Cancer Treatm Rev* **24**: 35–53

Rothenberg ML, Sharma A, Weiss GR, Villalona-Calero MA, Ecka Aylesworth C, Kraynak MA, Rinaldi DA, Rodriquez GI, Burris HA, Eckhardt SG, Stephens CD, Forral K, Nicol SJ and Von Hoff DD (1998) Phase I trial of paclitaxel and gemcitabine administered every two weeks in patients with refractory solid tumours. *Ann Oncol* **9**: 733–738

Rowinsky EK, Donehower RC, Jones RJ and Tucker RW (1988) Microtubule changes and cytotoxicity in leukemic cell lines treated with Taxol. *Cancer Res* **48**: 4093–4100

Rowinsky EK, Citardi MJ, Noe DA and Donehower RC (1993) Sequence dependent cytotoxic effects due to combinations of cisplatin and the antimicrotubule agents Taxol and vincristine. *J Cancer Res Clin Oncol* **119**: 727–733.

Rowinsky EK and Donehower RC (1995) Paclitaxel (Taxol). *New Eng J Med* **332**: 1004–1014

Ruiz van Haperen VWT, Veerman G, Boven E, Noordhuis P, Vermorken JB and Peters GJ (1994) Schedule dependence of sensitivity to 2′,2′-difluorodeoxycytidine (gemcitabine) in relation to accumulation and retention of its triphosphate in solid tumour cell lines and solid tumors. *Biochem Pharm* **48**: 1327–1339

Ruiz van Haperen VWT, Veerman G, Vermorken JB, Pinedo HM and Peters GJ (1996) regulation of phosphorylation of deoxycytidine and 2′,2′-difluorodeoxycytidine (gemcitabine): Effects of cytidine 5′-triphosphate and uridine 5′-triphosphate in relation to chemosensitivity for 2′,2′-difluorodeoxycytidine. *Biochem Pharm* **51**: 911–918

Sasvari-Szekely M, Spasokoukotskaja T, Szoke M, Csapo Z, Turi A, Szanto I, Eriksson S and Staub M (1998) Activation of deoxycytidine kinase during inhibition of DNA synthesis by 2-chloro-2′-deoxyadenosine (Cladribine) in human lymphocytes. *Biochem Pharmacol* **56**: 1175–1179

Sasvari-Szekely M, Csapo Z, Spasokoukotskaja T, Talianidis I and Staub M (1999) Modulation of human deoxycytidine kinase activity as a response to different cellular stresses. *Cell Mol Biol Lett* **Vol 4**: 462

Schiff PB, Fant J and Horwitz SB (1979) Promotion of microtubule assembly in vitro by paclitaxel. *Nature* **277**: 665–667

Seiter K, Feldman EJ, Traganos F, Li X, Halicka HD, Darzynkiewicz Z, Lederman CA, Romero MB and Ahmed T (1995) Phase I study on Taxol in acute leukemias: Evaluation of in vivo induction of apoptosis by Taxol. *Leukemia* **9**: 1961–1963

Spasokoukotskaja T, Arner ESJ, Brosjo O, Gunven P, Juliusson G, Liliemark J and Eriksson S (1995) Expression of deoxycytidine kinase and phosphorylation of 2-chlorodeoxyadenosine in human normal and tumour cells and tissues. *Eur J Cancer* **31**: 202–208

Theodossiou C, Cook JA, Fisher J, Teague D, Liebmann JE, Russo A and Mitchell JB (1998) Interaction of gemcitabine with paclitaxel and cisplatin in human tumour cell lines. *Intern J Oncol* **12**: 825–832

Tolis C, Peters GJ, Ferreira CG, Pinedo HM and Giaccone G (1999) Cell cycle disturbances and apoptosis induced by topotecan and gemcitabine on human lung cancer cell lines. *Eur J Cancer* **35**: 796–807

Torres K and Horwitz SB (1998) Mechanisms of Taxol-induced cell death are concentration dependent. *Cancer Res* **58**: 3620–3626

Van Moorsel CJA, Peters GJ and Pinedo HM (1997) Gemcitabine: future prospects of single agent and combination studies. *The Oncologist* **2**: 127–134

Van Moorsel CJA, Pinedo HM, Veerman G, Bergman AM, Kuiper CM, Vermorken JB, Van der Vijgh WJF and Peters GJ (1999) Mechanisms of synergism between cisplatin and gemcitabine in ovarian and non-small-cell lung cancer cell lines. *Br J Cancer* **80**: 981–990

© 2000 Cancer Research Campaign

Van Moorsel CJA, Bergman AM, Veerman G, Voorn DA, Ruiz van Haperen VWT, Kroep JR, Pinedo HM and Peters GJ (2000) Differential effects of gemcitabine on ribonucleotide levels of twenty-one solid tumour and leukemia cell lines. *Biochim Biophys Acta* **1474**: 5–12

Winter SF, Minna JD, Johnson BE, Takahashi T, Gazdar AF and Carbone DP (1992) Development of antibodies against p53 in lung cancer patients appears to be dependent on the type of p53 mutation. *Cancer Res* **52**: 4168–4174

*© 2000 Cancer Research Campaign*
